# Ai-Tong-An-Gao-Ji and Fisetin Inhibit Tumor Cell Growth in Rat CIBP Models by Inhibiting the AKT/HIF-1*α* Signaling Pathway

**DOI:** 10.1155/2022/1459636

**Published:** 2022-02-16

**Authors:** Jing Wang, Zonglang Lai, Xin Zhou, Song Na, Liufan Zhang, Jun Cheng

**Affiliations:** Department of Oncology, Chongqing Hospital of Traditional Chinese Medicine, Chongqing 400021, China

## Abstract

**Background:**

Ai-Tong-An-Gao-Ji (ATAGJ) has been extensively applied for acute bone cancer pain treatment with a satisfactory efficacy, while the specific mechanisms remain unclear and require further investigation.

**Methods:**

Overlapped genes of ATAGJ and CIBP obtained from SwissTargetPrediction website and GeneCards database were presented as a Venn diagram. A network diagram of drug-component-target was further established using the Cytoscape 3.6.0 software. The effect of fisetin on Walker 256 cell proliferation was observed by clone formation assay and EDU assay, and the interaction between fisetin and AKT was revealed using the immunoprecipitation assay. Effects of fisetin on AKT/HIF-1*α* signaling pathway in Walker 256 cells were ultimately detected using Western blot and qPCR assays.

**Results:**

The key component fisetin and core target gene AKT were sorted out using the drug-component-target network with a binding energy between fisetin and AKT less than −5 kcal/mol. Clone formation assay and EDU assay showed that fisetin substantially suppressed the proliferation of Walker 256 cells. Immunoprecipitation assay results revealed that the combination of fisetin and AKT decreased the level of AKT/HIF-1*α* signaling pathway of Walker 256 cells.

**Conclusions:**

The fisetin of ATAGJ can markedly suppress Walker 256 cells, and the mechanisms may be intimately associated with the combination of fisetin and AKT. Furthermore, fisetin decreased the level of p-AKT and inhibited the expression of the AKT/HIF-1*α* signaling pathway.

## 1. Introduction

As the treatment techniques for cancers advance, the five-year survival rate of cancer patients has been substantially improved. However, cancer-induced bone pain (CIBP) is ongoing and bothers the patients seriously, which greatly reduces their quality of life [1, 2]. Numerous studies have revealed that 55% of cancer patients and 66% of patients with advanced, metastatic, or terminal disease fall victim to CIBP [3].

CIBP represents the most common form of pain in cancer patients. About two-thirds of advanced cancer patients have a propensity to bone metastasis, which is reckoned to be a frequently encountered cause of cancer pain [4–6]. Currently, most strategies for CIBP treatment focuses on opioids, radiation therapy, and chemotherapy [7]. Unfortunately, the administration of opioids causes serious side effects, which often attenuates the therapeutic effect and the quality of life for cancer patients. A bunch of treatment methods based on traditional Chinese medicine including internal administration of decoction, external application, and acupuncture has achieved satisfactory clinical effects for cancer pain treatment. These therapeutic methods have the advantages of quick onset, safety, nontoxic side effects, and easy acceptance by patients [8, 9].

ATAGJ acts as effective preparation for CIBP management, and the main components of ATAGJ consist of borneol, spina gleditsiae, pillworm, faeces trogopterori, resina draconis, and semen strychni. The compound fisetin was contained in the spina gleditsiae, and it has been proved to play a role in antitumor by inhibiting tumor cell proliferation, inducing apoptosis, and mediating tumor cell migration [10–13].

Hypoxia-inducible factor-1*α* (HIF-1*α*) is a transcription factor at an extensive presence in mammals and humans under low oxygen levels. It responds to hypoxic tissue cells by elevating the expression of hypoxia-inducible genes, which represents the key link of adaptation to hypoxia. HIF-1*α* protein is highly expressed in most tumor tissues and the corresponding metastases. AKT pathway is a regulatory pathway of HIF-1*α*. AKT mainly regulates the changes of HIF-1*α* proteins [14–16].

We predicted active ingredients and related targets of cancer pain using the ointment in a network pharmacological approach. The targets of active ingredients and the target genes of CIBP were overlapped to identify core targets; GO analysis and KEGG pathway enrichment were subsequently conducted. The main components and core targets were selected for molecular docking. Additionally, whether fisetin acted on the proliferation and migration of Walker 256 cells were investigated by cloning, EDU, and transwell experiments, and the effects of fisetin on the AKT/HIF-1*α* signaling pathway Walker 256 cells were investigated by Western blot and qPCR assays.

## 2. Methods

### 2.1. Experimental Herbal Formulation

ATAGJ consists of borneol (BP), spina gleditsiae (ZJC), pillworm (SF), faeces trogopterori (WLZ), resina draconis (XJ), and semen strychni (MQZ). The ATAGJ administration dosage included low (10 g/d), medium (20 g/d), and high (30 g/d) doses. Female SD rats were employed as the laboratory animals and they were randomly classified into 5 groups: a sham group, a model group, a low dose ATAGJ group, a medium dose ATAGJ group, and a high dose ATAGJ group, with 10 rats in each group. The modeling procedures were described as follows: Walker 256 breast cancer cell lines were selected as the model cells. The rats were anesthetized with 0.3% sodium pentobarbital (1 ml/100 g). The white patellar ligament was exposed on the skin. The upper part of the tibia inferior to the white patellar ligament of the right hind limb was perforated. After the penetration into the bone marrow cavity, 4 *μ*L Walker 256 cell suspension at a concentration of 4 × 10^4^ cells/mL was injected into the model group. The rats were administered with low (10 g/day), medium (20 g/day), and high doses (30 g/day) of ATAGJ for 10 h and lasted for 14 d.

### 2.2. Cell Culture and Treatment

The Walker 256 breast cancer cell line was selected using 89% high glucose medium containing various amino acids and glucose (H-DMEM, High glucose Dulbecco's Modified Eagle Medium) + 10% fetal bovine serum (FBS) + 1% penicillin/streptomycin (P/S) [17].

Culture conditions were set at 37°C, 95% air, and 5% carbon dioxide. These cells were treated with fisetin (10 *μ*M, 20 *μ*M, and 30 *μ*M) and Cisplatin (5 *μ*M) in the positive control group for 24 h.

### 2.3. Network Pharmacology Analysis

Based on the principle of network pharmacology, the main components and targets of ATAGJ were predicted. The active ingredients of ATAGJ (BP, ZJC, SF, WLZ, XJ, and MQZ) were detected from the TCMSP website (https://tcmspw.com/tcmsp.php). Related targets were predicted and exported from the SwissTargetPrediction website. Human CIBP related genes were collected from the gene disease database, a PPI protein interaction network diagram was constructed using String, and the diagram network of drug-component-target was established using the Cytoscape 3.6.0 software. GO analysis and KEGG pathway enrichment were performed on 92 targets by Cytoscape ClueGO, and enrichment analysis results were visualized ultimately.

### 2.4. Molecular Docking

The 3D structure of fisetin was initially obtained from the TMMSP website. Meanwhile, the 3D structures of the key targets AKT and VEGFA were collected from the Protein Data Bank (https://www.rcsb.org/pdb). The AutoDock 4.2.6 software was used to hydrogenate the receptor protein and to calculate the charge treatment. The molecular docking between the receptor protein and the ligand small molecule was subsequently carried out by AutoDock Vina 1.1.2. The confirmation was obtained by docking and the binding energy was scored. The best binding energy was obtained and analyzed. PyMOL was used to visualize the interaction between the receptor protein and the ligand small molecule.

### 2.5. Paw Withdrawal Threshold (PWT)

The PWT of rats in each group was assessed every 7 days. During the process, the rats were put into a plexiglass cage equipped with a metal screen at the bottom for 5 min. The central skin of the hind paw at the molding side of the rats was vertically stimulated with Von Frey cilia mechanical stimulation probe so that the cilia were bent to the point where the rats had a paw constriction reflex. If there was no paw constriction reflex, a more intense cilia mechanical stimulation probe was replaced. Starting from 0.6 g, the stimulation of each intensity was 5 times and the mechanical stimulation interval was 15 s. The minimum ciliate stimulation probe strength was recorded as PWT with an upper limit of 15.0 g when 3 paw constriction reflexes occurred in the 5 tests [18].

### 2.6. Transwell Assay

The Transwell assay was performed to evaluate the capability of cell invasion. Cells of 6 × 10^4^ were initially followed by a cycle of washing with PBS and resuspended in 200 ml of serum-free medium. The upper Transwell chamber was precoated with Matrigel before the cells were supplemented. Simultaneously, the lower chamber was supplemented with a medium containing 10% FBS for incubation in 5% CO_2_ at 37°C for 24 h, followed by the addition of 4% paraformaldehyde (PFA) for fixation 15 min and stained for 3 min using crystal violet. Quantification was ultimately carried out using Axio Imager A2.

### 2.7. Clone Formation Assay

Cells at 1 × 10^3^ were planted in each well of 6-well plates and cultured at 37°C 5% CO_2_ for 14 d. The medium was refreshed every 3 days. Following two cycles of washing of the cell colonies using PBS, the cells were fixed with 4% paraformaldehyde for 30 min before being stained with 0.1% crystal violet for 20 min. Cell clone was triplicated three times.

### 2.8. EdU Assay

To perform the EdU assay, Walker 256 cells were inoculated into a 24-well plate. Following the instructions of the EdU kit, 2 × EdU reaction solution was prepared and added to a 24-well plate. Following incubation in the reaction solution for 2 h in the dark, the cells were fixed at room temperature for 20 min with 4% paraformaldehyde and added with 500 *μ*L 0.3% Triton X-100. PBS was subsequently added for rinsing 3 times when the reaction lasted 10 min at room temperature. AZIDE 555-Click reaction solution was subsequently prepared, 200 *μ*L of the solution was added to each well, and followed by incubation in the dark at room temperature for 30 min. The reaction solution was removed after three cycles of washing with PBS, and the nucleus was restained by Hoechst and then followed by the immunofluorescence technique.

### 2.9. Coimmunoprecipitation Assay

The kit used for biotin labeling fisetin was EZ-Link TM Biotin-LC-Hydrazide (Thermo Scientific). All procedures were carried out according to the operating instructions. The biotin-labeled fisetin was inoculated into Walker 256 cell suspension, and the cells were collected by centrifugation after 24 h of culture. Precooled coimmunoprecipitation assay A buffer was added. Cells were collected and centrifuged. The beads were washed twice with PBS, protein A agarose beads were added and centrifuged. Rabbit antibody was supplemented, and the antigen-antibody mixture was slowly shaken at 4°C overnight. Protein A agarose beads were subsequently added and shaken slowly at 4°C overnight. The agarose bead-antigen antibody complex was ultimately centrifuged, and electrophoresis was performed.

### 2.10. Western Blot Assay

Cells were collected and cleaved with immunoprecipitation assay lysate. After centrifugation at 13,000 rpm 4°C for 20 min, the supernatant was collected, and the total protein was separated utilizing 10% SDS-PAGE. Then the protein was transferred to a PVDF membrane, sealed with skimmed milk at room temperature for 1 h, and TBST was used to wash 3 times. Primary antibody was added for incubation at 4°C overnight, and second antibody was supplemented for incubation on the next day for 1 h. Finally, ECL color development was performed. The primary antibodies were listed below: anti-AKT, anti-p-AKT, anti-HIF-1*α*, anti-VEGF, and *β*-actin antibodies were from Cell Signaling Technology (Shanghai, China).

### 2.11. qPCR Assay

Total RNA was extracted using a Trizol reagent. Retranscription of the first cDNA strand was conducted using a Prime Scr immunoprecipitation assay kit [19]. When determining the relative expression level of genes, the reaction system and procedures of qPCR followed the instructions of the TB Green Premix TaqII. Relative expression levels of genes were measured and then calculated using 2^−∆∆CT^ algorithm methods.

### 2.12. Statistical Analysis

All data from the experiments were expressed as the mean ± standard deviation (SD). Student's *t*-tests were adopted for pairwise comparison and one-way analysis of variance (ANOVA) was for multiple group comparison. Statistical analysis was conducted using GraphPad Prism 7.0 software (LaJolla, CA, USA) and the differences were significant at *P* values < 0.05.

## 3. Results

### 3.1. GO and KEGG Analysis of CIBP Treated by ATAGJ

**Figure 1:**
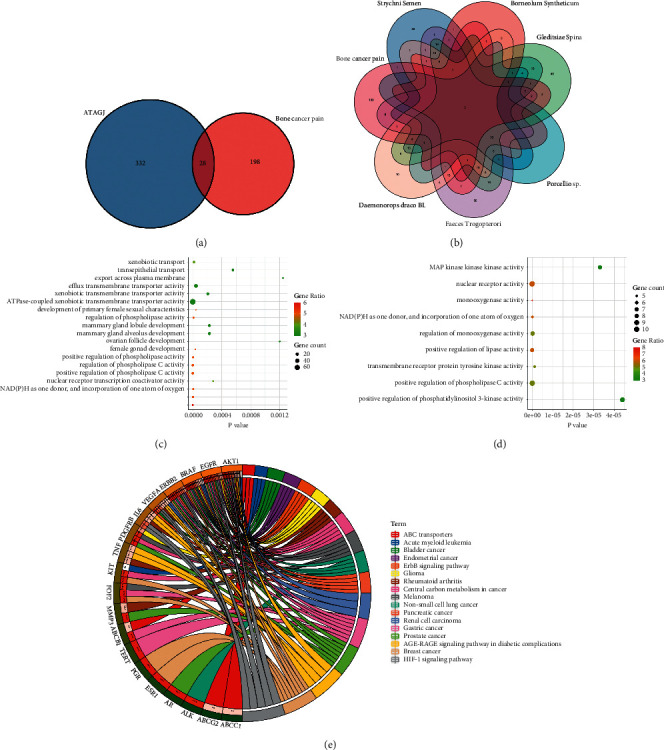
GO and KEGG pathway enrichment analysis; (a) Venn diagram of intersection gene between ATAGJ and bone cancer pain target; (b) gene venn diagram of intersection between borneol (BP), spina gleditsiae (ZJC), pillworm (SF), faeces trogopterori (WLZ), resina draconis (XJ) and semen strychni (MQZ), and bone cancer pain target. (c, d) Bubble diagrams of biological process and molecular function via GO analysis. The *Y* axis on the left is the name of the GO channel, and the *X* axis is the *P* value. The larger the circle is, the more genes are compared. The darker the color is, the more genes are compared. (e) KEGG pathway enrichment analysis circle diagram, the right side of the outermost layer is the names of the signaling pathways, and the left side is the genes. The left inner circle represents the significant *P* values of the pathways of the corresponding genes.

ATAGJ has been proved to be effective for CIBP through long-term clinical trials in this group. ATAGJ is composed of BP, ZJC, SF, WLZ, XJ, and MQZ. To investigate the effects of ATAGJ on CIBP, we first identified the active components and related targets of ATAGJ. There were 332 targets in ATAGJ, 198 targets in CIBP, and 28 overlapped targets (Figures 1(a) and 1(b)). To elucidate the function of ATAGJ targets and the role of potential targets in the signaling pathways, we analyzed the 28 targets utilizing GO and KEGG analysis and visualized the results of enrichment analysis. GO enrichment analysis revealed that the effects on eux transmembrane transporter activity, ATPase-coupled xenobiotic transmembrane transporter activity, NADPH as one donor, and incorporation of one atom of oxygen were more significant in biological processes. The effects of monooxygenase activity, nuclear receptor activity, positive regulation of phospholipase C activity in molecular function were more significant (Figures 1(c) and 1(d)). The results of the KEGG pathway analysis indicated that the 28 potential targets of ATAGJ for CIBP were positively related to the HIF-1*α* signaling pathway. Next, we verified the effect of ATAGJ on the HIF-1*α* signaling pathway (Figure 1(e)).

### 3.2. Component-Target Network Mapping and Molecular Docking in ATAGJ Treatment of CIBP

**Figure 2:**
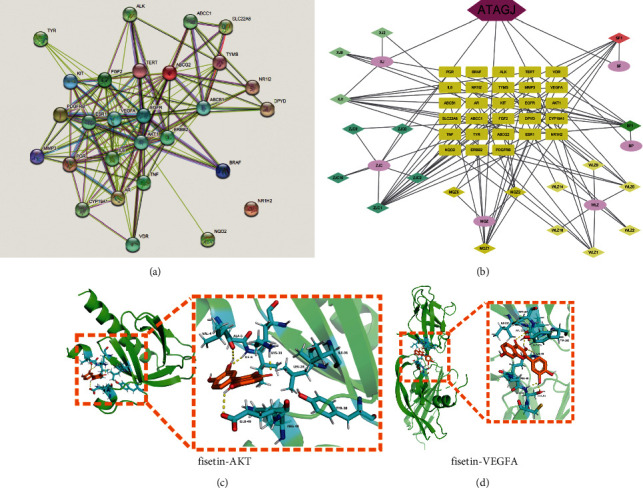
The key components and core targets of ATAGJ in treating bone cancer pain were analyzed by network pharmacology: (a) interaction analysis of 28 proteins; (b) drug-component-target gene network diagram. ATAGJ refers to compound MQZ, BP, ZJC, SF, WLZ, and XJ. MQZ refers to the borneol, spina gleditsiae, pillworm, faeces trogopterori, resina draconis, and semen strychni; (c) molecular docking between fisetin and AKT; (d) molecular docking between fisetin and VEGFA.

A network diagram of PPI protein interaction (Figure 2(a)) was construed via the String platform. We found that fisetin was one of the key components of ATAGJ, and AKT1 and VEGFA were the core target genes with high degree values (Figure 2(b)). The results showed that fisetin, AKT, and VEGFA were less than −5 kcal/mol. The amino acid residues ALA-5, ILE-6, and Glu-49 of AKT and fisetin formed hydrogen bond interaction and hydrophobic interaction with amino acid residues VAL-4, LYS-30, LEU-28, ILE-36, ARG48, and TYR38. The amino acid residues VAL-216, LYS-48, SER-50, and CYS-51 of VEGFA and fisetin formed hydrogen bond interaction and hydrophobic interaction with amino acid residues ILE-215, MET-197, TYR-165, and PRO-49. Molecular docking results showed that fisetin, the key component of ATAGJ, might affect CIBP by regulating AKT or VEGFA (Figures 2(c) and 2(d)). The KEGG pathway analysis revealed that the HIF-1*α* signaling pathway might play a pivotal role in treating CIBP following ATAGJ administration, and the AKT pathway was the regulatory pathway of HIF-1*α*. Therefore, we suspected whether ATAGJ and fisetin could regulate the HIF-1*α* signaling pathway by combining AKT.

### 3.3. ATAGJ Alleviates CIBP in Rats by AKT/HIF-1*α* Signaling Pathway

**Figure 3:**
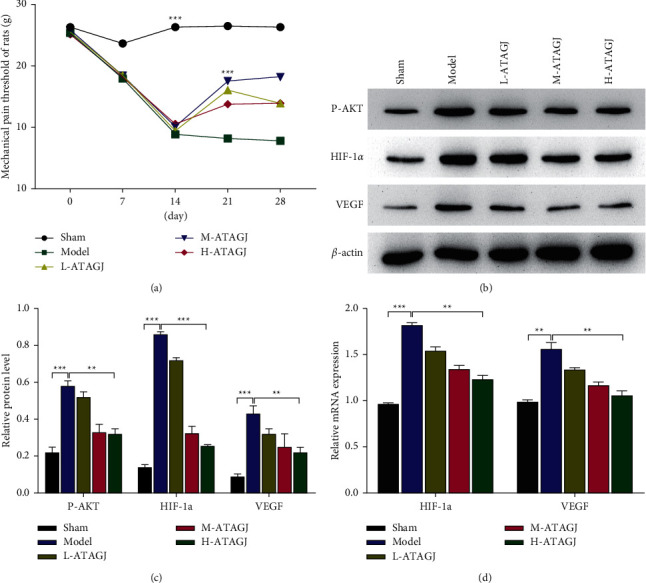
ATAGJ affects AKT/HIF-1*α* signaling pathway in CIBP rats; (a) mechanical pain threshold of rats in all groups; (b, c) western blot detection of protein expressions of p-Akt, HIF-1*α*, and VEGF. *β*-actin expression was regarded as an internal control. (d) qRT-PCR detection of mRNA expression levels of the indicated genes. Student's *t*-tests (two groups) or one-way ANOVA was employed and followed by Tukey's tests (more than two groups) (*n* ≥ 3).  ^*∗*^*P* < 0.05,  ^*∗∗*^*P* < 0.01, and  ^*∗∗∗*^*P* < 0.001.

Despite ATAGJ produced satisfactory clinical efficacy in patients with CIBP, its specific mechanism has not been defined yet. We analyzed CIBP in patients with possible AKT/HIF-1*α* signaling pathway regulation by ATAGJ in a network pharmacology approach. To figure out the molecular mechanism of ATAGJ affecting CIBP treatment, the SD rats were selected and divided into a sham group, a model group, a low dose ATAGJ group, a medium dose ATAGJ group, and a high dose ATAGJ group. At 9 a.m., ATAGJ was applied at low (10 g/d), medium (20 g/d), and high (30 g/d) doses, respectively, for 10 h and lasted for 14 d. The PWT of the model group was markedly lower than that of the sham group at day 7 (*P* < 0.01), which indicated that the pain threshold was decreased and the CIBP model was constructed successfully. No significant difference was exhibited between both groups (*P* > 0.05), which indicated that ATAGJ could substantially increase PWT and improve the pain threshold of the rats (Figure 3(a)). The effects of ATAGJ on the AKT/HIF-1*α* signaling pathway were subsequently detected using Western blot experiments, which suggested that the levels of p-AKT, HIF-1*α*, and VEGF were elevated markedly in the model group compared with the sham group (*P* < 0.01). The levels of p-AKT, HIF-1*α*, and VEGF decreased largely when compared with the model group (*P* < 0.01) (Figures 3(b) and 3(c)). The results of qPCR suggested that the levels of HIF-1*α* and VEGF rose substantially in the model group in contrast to those of the sham group (*P* < 0.01). Compared with the model group, the levels of HIF-1*α* and VEGF decreased significantly (*P* < 0.01) (Figure 3(d)).

### 3.4. ATAGJ's Monomer Fisetin Inhibits Tumor Growth

Fisetin was regarded as the key component (with the most connections) of ATAGJ through network pharmacology analysis. It is a compound derived from natural plants and characterized by a wide range of pharmacological effects. To investigate whether ATAGJ monomer fisetin affected the proliferation of Walker 256 cells, we classified Walker 256 cells into a control group, a cisplatin group, a low dose fisetin group, a medium dose fisetin group, and a high dose fisetin group. Walker 256 cells were treated with fisetin (10 *μ*M, 20 *μ*M, and 30 *μ*M) and Cisplatin (5 *μ*M) in the positive control group for 24 h.

**Figure 4:**
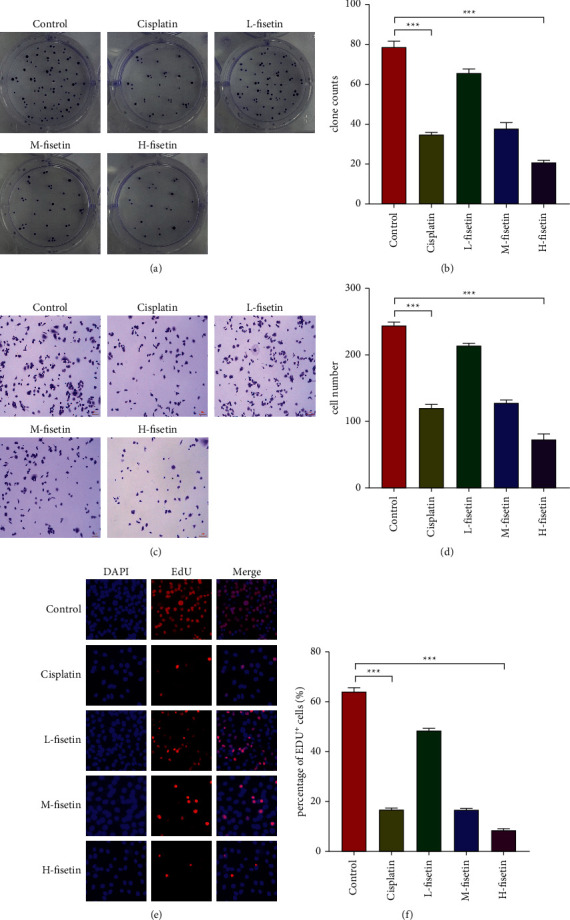
Fisetin affects tumor cell proliferation; (a) colony formation assay of each group of cells; (b) quantification of several colonies in Figure 4(a); (c) transwell experiment of cells in each group; (d) quantification of several cells in Figure 4(c); (e) EDU experiment of each group; (f) quantification of EDU^+^ cells in Figure 4(e). We divided the Walker 256 cells into a control group, cisplatin group, low dose fisetin group, medium dose fisetin group, and a high dose fisetin group. Walker 256 cells were treated with fisetin (10 *μ*M, 20 *μ*M, and 30 *μ*M) and cisplatin (5 *μ*M) in the positive control group for 24 h Student's *t*-tests (two groups) or one-way ANOVA was employed and followed by Tukey's tests (more than two groups) (*n* ≥ 3). ^*∗*^*P* < 0.05, ^*∗∗*^*P* < 0.01, ^*∗∗∗*^*P* < 0.001.

The results of the colony formation assay showed that the cisplatin group could apparently inhibit the proliferation of Walker 256 cells in both medium and high dose fisetin groups (*P* < 0.01) (Figures 4(a) and 4(b)). Further detection on the effects of fisetin was performed to identify the migration ability of Walker 256 cells by the Transwell experiment, indicating that the cisplatin group could substantially inhibit the migration of Walker 256 cells in both low and high dose fisetin groups (*P* < 0.01) (Figures 4(c) and 4(d)). Meanwhile, EDU experiment results showed that the cisplatin group could significantly inhibit the proliferation of Walker 256 cells in medium and high dose fisetin groups (*P* < 0.01) (Figures 4(e) and 4(f)).

### 3.5. ATAGJ Monomer Fisetin Inhibits Tumor Growth via AKT/HIF-1*α* Signaling Pathway

**Figure 5:**
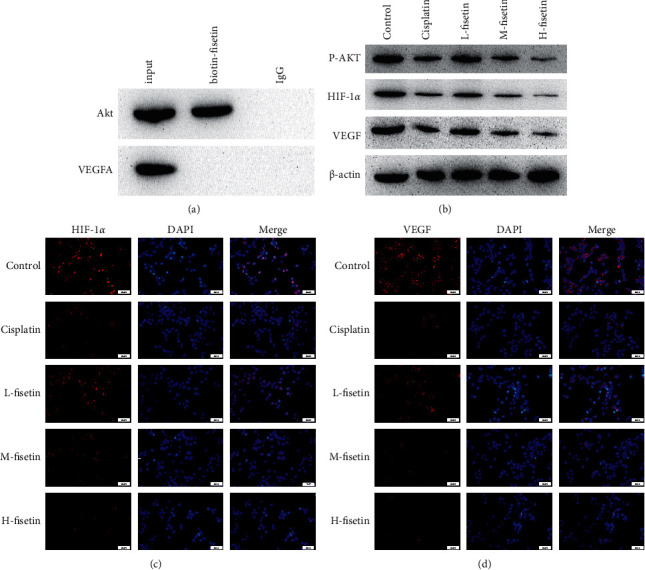
Fisetin affects the Akt/HIF-1*α* signaling pathway in tumor cells. (a) Coimmunoprecipitation assay showed fisetin-AKT interactions in Walker 256 cells. (b) Protein expressions of p-Akt, HIF-1*α*, and VEGF. (c, d) HIF-1*α* and VEGF were detected by immunofluorescence assay. Red represented HIF-1*α* and VEGF. Blue represented DAPI.

Through network pharmacology, we found that AKT1 and VEGFA were the core target genes with a high degree. KEGG pathway analysis showed that the HIF-1*α* signaling pathway might be of great importance in the treatment of CIBP by ATAGJ, and the AKT pathway was the regulatory pathway of HIF-1*α*. The results of molecular docking indicated that fisetin might regulate the HIF-1*α* signaling pathway by binding to AKT. To verify the relationship between fisetin and AKT1, we labeled fisetin with biotin to investigate whether this monomer could bind to AKT1. Co-IP results indicated that fisetin could be combined with AKT1 (Figure 5(a)). To investigate whether fisetin inhibited tumor growth through the AKT/HIF-1*α* signaling pathway, we found that fisetin could significantly reduce the levels of p-AKT, HIF-1*α*, and VEGF (*P* < 0.01) (Figure 5(b)). Meanwhile, fisetin was indicated to substantially decrease the levels of HIF-1*α* and VEGFR (*P* < 0.01) (Figures 5(c) and 5(d)).

## 4. Discussion

Cancer pain has been well-recognized as one of the common complications suffered by patients with a range of cancers, occurring approximately 25% for the first time [20]. The incidence of pain in advanced cancer patients can reach up to 60∼80%, and 1/3 of the patients suffer from ongoing severe pain. At present, most therapeutic methods preferred by western medicine in treating cancerous pain mainly include analgesic drugs, nerve block, primary lesion surgery, and chemoradiotherapy [9]. The treatment principles of drug analgesia are mainly based on the “third-order ladder” recommended by WHO [21]. Despite some effects that have been achieved in clinical practice, adverse reactions including gastrointestinal reaction, constipation, vertigo, respiratory depression, and mental disorder are also present [22]. It is, therefore, an urgent need to find a satisfactory therapy that can win the confidence of cancer pain patients.

ATAGJ functions as an effective therapeutic option for CIBP relief and pain management. Its main components consist of borneol, spina gleditsiae, pillworm, faeces trogopterori, resina draconis, and semen strychni. Spina gleditsiae contains the compound fisetin, which represents a kind of yellow bioactive pigment [23]. The molecular formula of fisetin is C_15_H_10_O_6,_ with a molar mass of 286.2363 g/mol and a density of 1.688 g/mL. It is soluble in ethanol, acetone, acetic acid, and hydroxide base solution [24, 25]. In general, some pain inducing mediators including tumor cells and inflammatory cells are reckoned to be involved in the occurrence and development of CIBP. Also, continuous activation of osteoclasts is considered to be related to this pain. Furthermore, tumor expansion on the nerve compression and damage is also a source of pain [26]. Tumors are not highly dominated by sensory neurons. However, rapid tumor growth has a propensity to bind to and damage nerves, resulting in mechanical damage, compression, ischemia, or direct proteolysis. As the proliferation of tumor cells, they first compress then destroy the hematopoietic cells that normally make up the bone marrow and the sensory fibers that normally dominate the bone marrow and mineralized bone [1].

We initially identified the active components and related targets of ATAGJ by network pharmacology. And the GO analysis and KEGG pathway analysis were performed using the Cytoscape ClueGO plugin. The findings of the KEGG pathways revealed that the 28 potential targets of ATAGJ treatment for CIBP were mainly correlated to the HIF-1*α* signaling pathway. HIF-1*α* was the core regulator of inducing hypoxia gene and intracellular oxygen environment repair, which could regulate cell growth, proliferation, migration, inflammation, and apoptosis. High expression of HIF-1*α* protein was indicated in most tumor tissues and their metastatic sites [14–24, 27–29]. Then Zhang et al. reported that CIBP was alleviated through inhibiting the HIF-1*α*/vascular endothelial growth factor signaling pathway [30]. Therefore, we concluded that ATAGJ might reduce CIBP by inhibiting the expression of HIF-1*α*. We then validated the hypothesis by constructing a CIBP model of rats. The mechanical pain thresholds of the low, middle, and high dose ATAGJ groups were higher than that of the model group at day 21 (*P* < 0.01). No significant difference was exhibited between both groups (*P* > 0.05), which indicated that ATAGJ had a satisfactory effect on reducing CIBP. Furthermore, we observed that the expression levels of p-AKT, HIF-1*α*, and VEGF were markedly elevated in the model group instead of sham group (*P* < 0.01). Compared with the model group, those of p-AKT, HIF-1*α*, and VEGF decreased greatly in H-ATAGJ treatment group (*P* < 0.01). The results suggested that ATAGJ could reduce CIBP in rats by reducing AKT/HIF-1*α* signaling pathway. We visualized the drugs, components, and targets using the Cytoscape 3.6 software and found that fisetin was the key component in ATAGJ (with the most connections). It indicated that the fisetin group could markedly inhibit the proliferation of Walker 256 cells in both low and high dose fisetin groups (*P* < 0.01). Further, we analyzed whether fisetin acted on the migration of Walker 256 cells by the Transwell experiment. Significant inhibition of the fisetin group on the migration of Walker 256 cells was revealed in medium and high dose fisetin groups (*P* < 0.01). Concomitantly, the EDU experiment indicated that the fisetin group could markedly inhibit the proliferation of Walker 256 cells in medium and high dose fisetin groups (*P* < 0.01). The previously described results indicated that fisetin could greatly suppress the proliferation and metastasis of tumor cells.

AKT1 and VEGFA are the core target genes with high degree scores. The results of molecular docking indicated that fisetin, AKT, and VEGFA were less than −5 kcal/mol. We, therefore, speculated that fisetin might affect CIBP by regulating AKT or VEGFA. We then labeled fisetin with biotin to investigate whether it could bond to AKT1. Co-IP results revealed that fisetin could be combined with AKT1. From this, we hypothesized fisetin could regulate the HIF-1*α* signaling pathway by binding to AKT. In addition, we found that fisetin could significantly reduce the levels of HIF-1*α*, p-AKT, and VEGF (*P* < 0.01). This suggested that fisetin inhibited the AKT/HIF-1*α* signaling pathway in tumor cells by binding to AKT.

## 5. Conclusion

We initially predicted the potential targets and pathways of ATAGJ for CIBP management using a network pharmacology approach. The clone formation and proliferation of Walker 256 cells were detected after fisetin treatment. Furthermore, experiments were performed to detect the AKT/HIF-1*α* signal pathway expression in CIBP rats and Walker 256 cells. The molecular docking and IP experiments verify the binding of fisetin and AKT. The results demonstrated the effect of ATAGJ in CIBP rats and the key component fisetin could suppress Walker 256 cells proliferation and downregulate the expressions levels of HIF-1*α*, p-AKT, and VEGF through targeting AKT.

## Data Availability

The data used to support the findings of this study are included within the article.
